# Peripheral B-Cell Subset Distribution in Primary Antiphospholipid Syndrome

**DOI:** 10.3390/ijms19020589

**Published:** 2018-02-16

**Authors:** Lorena Álvarez-Rodríguez, Leyre Riancho-Zarrabeitia, Jaime Calvo-Alén, Marcos López-Hoyos, Víctor Martínez-Taboada

**Affiliations:** 1 Transplantation and Autoimmunity Laboratory, Rheumatology Department, University Hospital Marqués de Valdecilla-IDIVAL, 39008 Santander, Spain; lorenalvar@hotmail.com; 2 Rheumatology Department, Hospital Sierrallana, 39300 Torrelavega, Spain; leyre1987@hotmail.com; 3 Rheumatology Department, University Hospital Araba, 01004 Vitoria, Spain; jcalvo@ser.es; 4 Immunology Department, University Hospital Marqués de Valdecilla-IDIVAL, Faculty of Medicine, Cantabria University, 39008 Santander, Spain; mlopezhoyos@humv.es; 5 Rheumatology Department, University Hospital Marqués de Valdecilla-IDIVAL, Faculty of Medicine, Cantabria University, 39008 Santander, Spain

**Keywords:** APS, SLE, B cell, BAFF, autoimmunity, IL-6, inflammation

## Abstract

Background: B-cell differentiation and B-cell tolerance checkpoints may be different in antiphospholipid syndrome (APS) from systemic lupus erythematosus (SLE) and can help to understand differences between them. Our aim was to define alterations of B-cell subsets in patients with primary APS (pAPS) and to compare them with SLE patients and healthy controls (HC). Methods: Cross-sectional study including three study groups: 37 patients with pAPS, 11 SLE patients, and 21 age- and gender-matched HC. We determined the frequencies of different B-cell subsets in peripheral blood naïve and memory compartments. In addition, we measured serum B cell-activating factor (BAFF) levels and circulating pro-inflammatory cytokines, such as IL-6, by commercial ELISA and CBA, respectively. Results: Patients with pAPS showed a lower percentage of immature and naïve B cells than patients with SLE (*p* = 0.013 and *p* = 0.010, respectively) and a higher percentage of non-switched memory B cells than patients with SLE (*p* = 0.001). No differences either in the percentage of switched memory cells or plasma cells were found among the different groups. Serum BAFF levels were higher in SLE patients than in healthy controls and pAPS patients (*p* = 0.001 and *p* = 0.017, respectively). A significant increase in the serum BAFF levels was also observed in pAPS patients compared to HC (*p* = 0.047). Circulating IL-6 levels were higher in SLE and pAPS patients than HC (*p* = 0.036 and *p* = 0.048, respectively). A positive correlation was found between serum BAFF and IL-6 levels in patients with SLE but not in pAPS (*p* = 0.011). Conclusions: Our characterization of peripheral blood B-cell phenotypes in pAPS demonstrates different frequencies of circulating B cells at different stages of differentiation. These differences in the naïve B-cell repertoire could explain the higher number and variety of autoantibodies in SLE patients in comparison to pAPS patients, especially in those with obstetric complications.

## 1. Introduction

Antiphospholipid syndrome (APS) is a systemic autoimmune disease characterized serologically by the presence of antiphospholipid antibodies (aPL) and at least one clinical event defined as vascular thrombosis and/or pregnancy morbidity [1]. aPL are a heterogeneous group of autoantibodies, which includes lupus anticoagulant (LA), immunoglobulin (Ig) G and IgM anti-cardiolipin antibodies (aCL), and anti-β2 glycoprotein I (anti-β2GPI) antibodies. Heterogeneous aPL can disrupt physiological cellular functions through the activation of endothelial cells, thrombocytes, and placental tissue, leading to thrombosis and obstetric complications [2]. Several lines of evidence indicate that aPL contribute to inflammation, suggesting that the inflammatory response plays an important role in the pathogenesis of aPL-mediated diseases [3,4]. Thus, aPL have also emerged as triggers of innate immune inflammatory pathways in APS [5]. Although a clear association between aPL and clinical manifestations has been established, the pathogenesis of this syndrome is poorly understood [6].

Alterations of B-cell activation and B-cell subsets contribute to the development of autoimmune diseases, such as rheumatoid arthritis (RA), type 1 diabetes (T1D), Sjögren syndrome (SS), multiple sclerosis (MS), and systemic lupus erythematosus (SLE) [7,8,9,10]. Disturbances in B-cell homeostasis associated with defective functions have been also described in APS [11,12], although the exact functions of B-cell subsets and plasma cells remains largely unknown [13].

APS is a humoral autoimmune disorder that may share some similarities with other systemic autoimmune rheumatic diseases, such as SLE, but with unique characteristics, such as a specific vascular pathology and a relatively restricted antibody repertoire. It is possible that B-cell differentiation and B-cell tolerance checkpoints are different in APS and SLE and can help to understand differences between both disorders. The main three early checkpoints to remove autoreactive B cells before entering the mature memory B-cell compartments occur, first, in the bone marrow at the immature stage, second, at the transition to naïve B cells, and third, just before entering the germinal centre. Thus, a deregulation at any of these three checkpoints can lead to both an altered naïve B cell repertoire(BCR) repertoire and the generation of autoantibody-producing B cells.

The cytokine B cell-activating factor (BAFF) has an important role in B-cell survival and homeostasis [14] and its overexpression promotes loss of B-cell tolerance [15,16]. An increase of serum BAFF levels has been implicated in the development of SLE and other autoimmune diseases [17], but the precise role for this cytokine in the pathogenesis of APS has yet to be elucidated. In addition to its effect on B-cell survival, BAFF can also activate monocytes to produce IL-6 [18]. Our previous studies based on gene expression analysis demonstrated a monocyte activation in PBMCs from primary APS (pAPS) patients compared with those from healthy controls (HC) or SLE patients [19]. 

In the present work, we analyzed in peripheral blood the frequencies of different B-cell subsets and soluble factors in patients with pAPS and compared them with SLE patients and healthy controls (HC). We studied an SLE cohort as the disease control group, since changes in peripheral blood B-cell subsets have been extensively described and can help to validate our results in pAPS where data are scarce and somewhat contradictory.

## 2. Results

### 2.1. Patients with pAPS Differ in the B-Cell Repertoire Compared to Patients with SLE

To study possible alterations of B-cell subsets, we first determined the absolute number of peripheral blood CD19^+^ B cells in the peripheral blood of both patients and healthy controls. There were no significant differences in the circulating B cells among the different groups (226.5 cells/mm^3^ (181.8–341.3) versus 185 cells/mm^3^ (140.5–296) versus 112 cells/mm^3^ (87–325) in HC, pAPS, and SLE respectively, *p* = 0.184) (Table 1).

**Figure 1:**
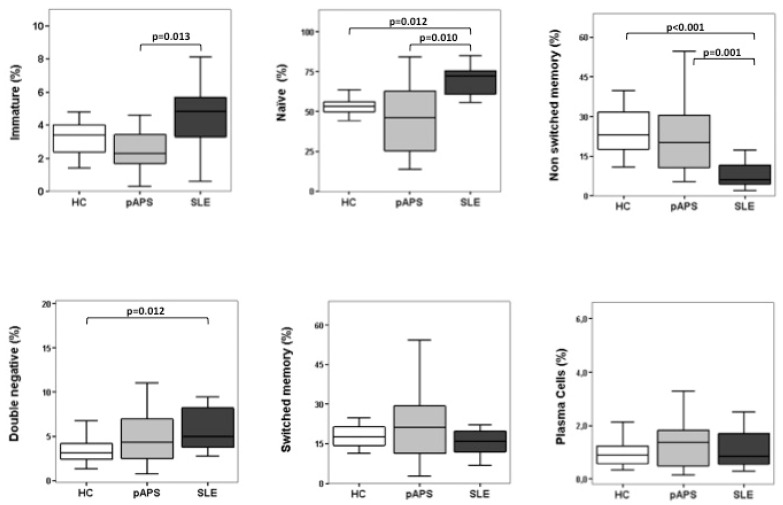
B-cell differentiation subsets in patients with primary antiphospholipid syndrome (pAPS), patients with systemic lupus erythematosus (SLE), and healthy controls (HC). Percentages of different B-cell subsets: transitional-immature (CD19^+^CD5^+^CD10^+^ CD27^−^IgD^++^ CD24^hi ^CD38^hi^), naïve (CD19^+^ CD5^+/−^ CD10^−^ CD27^−^IgD^+^ CD24^int ^CD38^low^/^−^), non-switched memory (CD19^+^ CD27^+ ^IgD^+^ CD38^−^ IgM^+^), double-negative (CD19^+^ CD27^−^IgD^−^ CD24^−^ CD38^−/+ ^IgM^−^), switched memory (CD19^+^ CD27^+^IgD^−^ CD38^+^IgM^+/−^), and plasma cells(CD19^lo^ CD27^hi^IgD^−^ CD38^hi ^CD138^−/+^) in HC and in patients with pAPS or SLE. CD19^+^ cells were gated for analysis. Box plots show the median and interquartile range. Differences were significant when *p* value <0.05 by Mann–Whitney U test. HC: healthy controls; pAPS: primary antiphospholipid syndrome; SLE: systemic lupus erythematosus.

**Figure 2:**
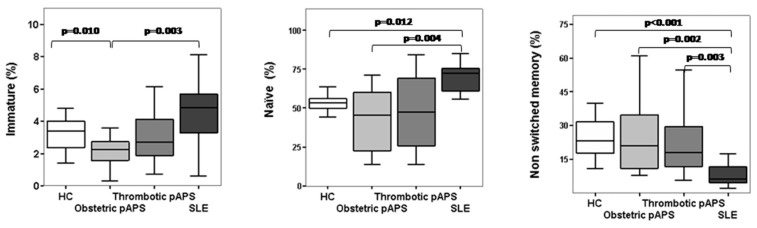
B-cell differentiation subsets in patients with obstetric and thrombotic primary antiphospholipid syndrome (pAPS). Percentages of transitional-immature (CD19^+^CD5^+^CD10^+^ CD27^−^IgD^++^ CD24^hi^ CD38^hi^), naïve (CD19^+^ CD5^+/−^ CD10^−^ CD27^−^IgD^+^ CD24^int ^CD38^low^/^−^), and non-switched memory (CD19^+^ CD27^+^IgD^+^ CD38^−^IgM^+^) B cells in patients subdivided into obstetric and thrombotic pAPS and compared to HC and SLE. Box plots show the median and interquartile range. Differences were significant when *p* value <0.05 by Mann–Whitney U test. Abbreviations: HC, healthy controls; pAPS, primary antiphospholipid syndrome; SLE, systemic lupus erythematosus.

Regarding the percentage of circulating B-cell subsets at different checkpoints (immature, naïve, non-switched memory, switched memory, double-negative, and plasma cells), no significant differences were found either in the frequencies of immature or naïve B cells between pAPS patients and HC. However, we found that the frequencies of immature and naïve B cells were significantly decreased in pAPS patients compared to SLE patients. Instead, the frequencies of non-switched memory B cells were significantly increased in pAPS compared to SLE (Figure 1). Interestingly, when we subdivided the pAPS group according to clinical manifestations, those with obstetric APS showed significantly decreased frequencies of immature B cells compared to those with SLE and HC (*p* = 0.003 and *p* = 0.010, respectively) and decreased frequencies of naïve B cells compared to those with SLE (*p* = 0.002) (Figure 2). These differences were not observed in thrombotic APS. Additionally, not statistically significant changes were found between thrombotic and obstetric APS. The frequencies of non-switched memory B cells were increased both in obstetric and thrombotic pAPS compared to SLE (*p* = 0.002 and *p* = 0.003, respectively).

On the other hand, patients with SLE had an increased frequency of naïve B cells as well as a decreased frequency of non-switched memory B cells compared to HC. In addition, a significant increase in the percentage of double-negative B cells was found in SLE patients as compared with HC (*p* = 0.012) but not with pAPS patients (Figure 1). Finally, pAPS showed a slight increase, although no differences were found either in the percentages of circulating switched memory cells or plasma cells among the three groups (Figure 1).

### 2.2. High Serum BAFF Levels Correlate with IL-6 in Patients with SLE but Not in pAPS Patients

**Figure 3:**
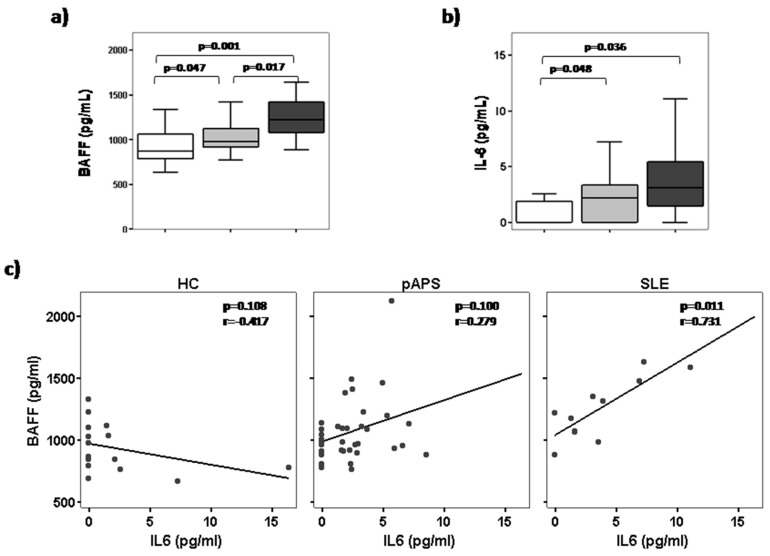
Serum B cell-activating factor (BAFF) and IL-6 levels in patients with primary antiphospholipid syndrome (pAPS), patients with systemic lupus erythematosus (SLE), and healthy controls (HC). Correlation between BAFF and IL-6 levels. Serum levels of BAFF (**upper left**) and IL-6 (**upper right**) were determined by ELISA and cytometric bead array, respectively, in HC, pAPS, and SLE. Box plots show the median and interquartile range. Differences were significant when *p* value <0.05 by Mann–Whitney U test. Correlation dot plots between serum BAFF levels and IL-6 levels in HC, pAPS, and SLE (**low panel**)**.** The Spearman correlation coefficient and the level of significance are shown in the upper-right corner of each plot. The fit line is also displayed.

Since BAFF plays an important role in B-cell survival and homeostasis, and considering our results, we measured serum BAFF levels in the same patients in whom we determined circulating B-cell subsets frequencies. SLE patients showed significantly higher serum BAFF levels than pAPS patients and HC (*p* = 0.017 and *p* = 0.001, respectively; (Figure 3A). Also, slightly increased serum BAFF levels were found in pAPS patients compared to HC (*p* = 0.047).

Apart from its effects on B cells, BAFF can also activate monocytes to produce IL-6 [18]. Thus, we also analysed the circulating IL-6 levels and we observed that pAPS and SLE patients had higher IL-6 levels than HC (*p* = 0.036 and *p* = 0.048, respectively), but no differences were found between APS compared to SLE (Figure 3B). Obstetric and thrombotic APS displayed similar IL-6 levels. A positive correlation (*p* = 0.011, *r* = 0.731) was found between serum BAFF levels and IL-6 levels in patients with SLE but not with pAPS (Figure 3c).

## 3. Discussion

B cells are thought to be crucial in the pathogenesis of APS, since it is an autoantibody-mediated disease; however, there are only a few small-sized studies addressing B-cell homeostasis in APS [11,12,20] (Table S1).

We investigated whether the frequency of circulating B-cell subsets in patients with pAPS differed from that in patients with SLE and healthy controls. We included 37 patients with defined pAPS (17 obstetric pAPS and 20 thrombotic pAPS) that showed a lower percentage of immature and naïve B cells than SLE patients and a higher percentage of non-switched memory cells than SLE patients. These differences were mainly due to patients with obstetric complications. We found no differences either in the percentage of switched memory cells or in plasma cells among the different groups.

Discordant results have been published regarding the circulating CD19^+^ B cells in APS. A decrease in the number of CD19^+^ B cells in pAPS [20] and in SLE-secondary APS were reported [21]. However, other studies did not observe differences in the frequency of peripheral blood CD19^+^ B cells in pAPS without thrombosis or with venous thromboembolism (VTE) compared to controls [11,12]. In agreement with these latter findings, we did not find any differences either in the absolute number or in the percentage of CD19^+^ B cells in pAPS compared to HC.

Carbone et al. [11] studied 36 women with obstetric APS (10 of them also with thrombosis) and compared them with controls. They found that only patients with thrombosis had increased percentages and absolute numbers of naïve B cells compared to APS without thrombosis and controls. Likewise, an increase in the number but not in the frequency of naïve B cells was reported in pAPS-VTE compared to non-APS-VTE but not to HC [12]. They also reported a decrease in the number and percentage of switched memory B cells in pAPS patients with thrombotic complications. In contrast, our study did not demonstrate differences in the frequencies of any peripheral blood B cell subsets in pAPS compared to HC, except a significant decreased frequency of immature B cells in obstetric pAPS. Moreover, patients with thrombotic and obstetric APS showed a trend to have lower percentages of naïve B cells than HC.

There is only scarce information comparing B-cell distribution in pAPS and SLE. Simonin et al. [12] compared 11 VTE-pAPS with other autoimmune diseases, including 11 SLE patients. VTE-pAPS patients showed higher number of naïve B cells and a reduction in the non-switched and switched memory B cells compared with SLE. Unlike them, our results indicate lower percentages of naïve cells in pAPS than in SLE and a higher number of non-switched memory cells in pAPS.

The differences of our results with others may be due to several reasons. First of all, the groups included in both studies were clinically different. Carbone et al. [11] studied 36 women with pAPS (with or without thrombosis). Simonin et al. [12] studied 11 pAPS with venous thrombosis, but without information about obstetric morbidities or arterial thrombosis. Moreover, they compared them with 11 SLE patients, not reporting information about disease activity.

We also recognize that the results of the present study might be limited for several reasons. The first reason is the selection of the study population. Only SLE patients with low disease activity (SLEDAI ≤4) were included to avoid possible influence of the systemic inflammatory component of the disease in B-cell distribution. Secondly, the influence of the different treatments, both for SLE and pAPS, were not taken into account. Mainly, pAPS patients were treated with antiplatelet and/or anticoagulant therapies, and these treatments have not been found up to now to have an influence on peripheral blood circulating B-cell subsets. Patients with SLE were mainly treated with chloroquine and corticosteroids. Chloroquine has been found to decrease serum BAFF levels and B-cell activation in SLE, although we observed increased BAFF levels. Finally, the time-point of the study in pAPS might also be relevant. This may be especially true in obstetric APS, where we have recently described that a significant percentage of patients lose their aPL during long-term follow-up [22]. On the other hand, the present study has some strengths. Our cohort of pAPS patients is probably the largest compared with others (Table S1) with a compensated distribution of both subtypes of APS, thrombotic and obstetric. Nonetheless, comparisons between HC and pAPS reached a statistical power of 72.45%. In addition, the characterization of B-cell subsets has been performed by a six-colour flow cytometry analysis, which allows for a better characterization of B-cell subsets.

Several disturbances in B-cell homeostasis have been described thoroughly in SLE. Previous studies reported a significantly higher percentage of immature transitional cells (CD19^+^CD38^hi^ CD24^hi^) [23,24,25,26,27,28] in patients with SLE compared to HC. Moreover, Sanz et al. [25] demonstrated an expansion of double-negative (IgD^−^CD27^−^) memory B cells as well as a large reduction in the frequency of non-switched memory B cells (IgD^+^CD27^+^) in the peripheral blood of SLE patients compared to controls. Our data are in line with such studies, since we also found a significant increase in the percentage of naïve and double-negative B cells as well as a decreased frequency of non-switched memory B cells in SLE patients compared to controls. Furthermore, although it did not reach statistical significance, we observed a trend to have higher percentages of immature transitional B cells in SLE patients compared to HC. Interestingly, in addition to these differences to healthy controls, SLE patients showed a higher frequency of immature and naïve B cells, but a lower frequency of non-switched memory B cells, than pAPS patients.

During a physiological immune response, most B cells generated are autoreactive and are removed at different stages of B-cell development through mechanisms of immunological tolerance (reviewed by Nemazee et al.) [29]. Additionally, dysregulation in the B cells leads to an increase in autoreactive B cells leading to autoimmunity, as previously reported in patients with SLE and other autoimmune conditions [10,15,30]. Apart from an impaired deletion of autoreactive B cells, an excess of survival factors in the milieu, such as the cytokine BAFF, could promote the persistent presence of self-reactive B cells in the periphery of SLE patients [31,32]. The BAFF system plays a key role as a B cell-survival factor and is essential for the maturation of B lymphocytes, but it is also involved in the development of humoral autoimmunity, especially in SLE [33,34]. Probably, increased BAFF levels result in relaxed selection and can rescue low-affinity self-reactive transitional cells, thereby promoting the development of an altered repertoire of self-reactive naïve B cells (reviewed by Rawlings et al.) [17,35,36]. As in previous studies [37,38,39,40], we found increased serum BAFF levels in SLE patients compared to HC and pAPS patients.

Autoreactive B cells need to be activated to induce autoantibody production [41]. Thus, BAFF can also activate monocytes to produce IL-6 [18] and other pro-inflammatory cytokines that finally help to activate autoreactive B cells. Inflammation is not the most prominent characteristic of APS, but clinical and experimental data indicate that inflammatory processes are involved in the pathogeny of the syndrome [3]. Our previous study demonstrated that monocytes from pAPS are pro-inflammatory and secrete higher inflammatory cytokines than HC [19]. Further experiments are needed to demonstrate a possible link between BAFF levels and IL-6 production in SLE and APS patients.

Taken together, our data support changes in circulating B cell subsets in SLE patients but not in pAPS patients. Particularly, differences in the naïve B-cell repertoire could explain the higher number and variety of autoantibodies in SLE in comparison to pAPS, especially in those with obstetric complications.

## 4. Materials and Methods

### 4.1. Subjects

This cross-sectional study included three groups of Caucasian patients: pAPS, SLE, and HC. We studied 37 patients with pAPS (17 patients with obstetric complications and 20 patients with thrombotic phenomena). They all met the Sidney classification criteria [1]. Anti-cardiolipin IgG and IgM antibodies (aCL) and anti-beta 2 glycoprotein I IgG and IgM antibodies (anti-B2GPI) used for the criteria were determined in serum with commercial ELISA (Aesku Diagnostics, Wendelsheim, Germany). Lupus anticoagulant (LA) was determined in plasma in accordance with International Standards [1]. The laboratory parameters were confirmed at least in two samples separated by at least 12 weeks [1] and measured after clinical events. None of the APS patients had a clinical event related to the disease within the previous year of blood sampling for the study. We included, as the disease control group, a cohort of 11 patients with SLE that fit ACR 1997 and SLICC 2012 classification criteria [42,43]. All SLE patients were in remission or presented low disease activity defined by a score of <4 in the systemic lupus erythematosus disease activity index (SLEDAI). As a control group, we included 21 healthy controls (HC), with equivalent mean age and gender frequencies, without a previous history of infectious, neoplastic, or autoimmune disease. The main demographic, clinical, therapeutic, and laboratory characteristics of the study population are shown in Table 1. This study was approved by the Regional Ethics Committee (CEIC de Cantabria; approval date: 1 September 2007; code number 2007.108). All subjects gave signed informed consent in accordance with the Declaration of Helsinki.

### 4.2. Quantification of B Cells

Absolute numbers of peripheral blood CD19^+^ B cells were determined according to the MultitestTruCount method, as described by the manufacturer (BD Biosciences, San Jose, CA, USA). Data were acquired using FACScalibur (BD Biosciences) and analyzed with Multiset^TM^ software (BD Biosciences).

### 4.3. Circulating B Cells Immunophenotyping

**Figure 4:**
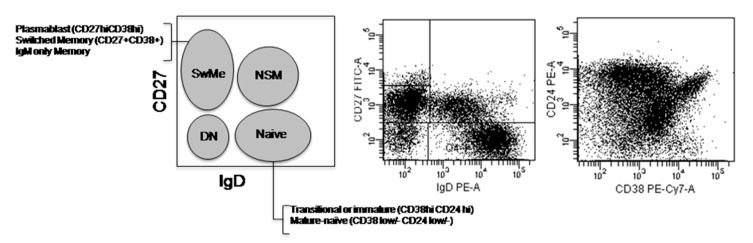
Circulating B cells immunophenotyping. Flow cytometry gating strategy to classify human B-cell subsets mainly according to the expression of CD27/IgD and CD24/CD38 within peripheral blood CD19^+^ B cells. DN: double-negative; NSM: non-switched memory; SwME: switched memory.

Peripheral blood mononuclear cells (PBMCs) from heparinized peripheral blood were obtained by Ficoll Histopaque 1077 (Sigma Aldrich, St. Louis, MI, USA) gradient centrifugation. Briefly, PBMCs were stained on surface with the following specific fluorochrome-conjugated monoclonal antibodies: anti-CD27 FITC (clone M-T-271), anti-CD24 FITC and PE (clone ML5), anti-IgD PE (clone A6-2), anti-CD5 PerCP Cy5.5 (clone L17F12), anti-CD138 PerCP Cy5.5 (clone MI15), anti-CD10 APC (clone HI10a), anti-IgM APC (clone G20-127), and anti-CD19 APC-Cy7 (clone SJ25C1) (all of them from BD Biosciences) and anti-CD38 PECy7 (clone HIT2) (Biolegends, San Diego, CA, USA). The immunophenotypic characterization of human B-cell subsets primarily relied on the expression of surface markers, such as CD19, IgD, CD27, CD24, and CD38, by flow cytometry as previously reported [25] (Figure 4). Other markers were added to better define populations, so the main B-cell subsets identified were (Table S2): immature (CD19^+^CD5^+^CD10^+^ CD27^−^IgD^++^ CD24^hi ^CD38^hi^), naïve (CD19^+^ CD5^+/−^ CD10^−^ CD27^−^IgD^+^ CD24^int ^CD38^low^/^−^), non-switched memory (CD19^+^ CD27^+^IgD^+^ CD38^−^ IgM^+^), switched memory (CD19^+^ CD27^+^IgD^−^ CD38^+^IgM^+/−^), double-negative (CD19^+^ CD27^−^IgD^−^ CD24^−^ CD38^−/+^IgM^−^), and plasma cells (CD19^lo^ CD27^hi^IgD^−^ CD38^hi^CD138^−/+^). Cells were acquired in a FACS Canto II Flow Cytometer (BD Biosciences) and data were analyzed using FACSDiva.

### 4.4. Serum BAFF Measurement

The serum was isolated from 4 mL of blood obtained in tubes without additives from each individual and stored at −80 °C until analysis. Serum levels of BAFF were determined using the commercial human BAFF Quantikine enzyme-linked immunosorbent assay (ELISA) Kit (R&D System; Minneapolis, MN, USA) according to the manufacturer’s instructions. The minimum detectable level of BAFF was 2.68 pg/mL.

### 4.5. Detection of Soluble Cytokine in Serum

The quantitative determination of IL6 in serum was performed using the Cytometric Bead Array (CBA) Human Inflammation kit (BD Biosciences). The fluorescence produced by CBA beads was measured on a FACS Canto II Flow Cytometer (BD Biosciences) and analyzed using FCAP array software (Soft Flow Inc.; New Brighton, MN, USA). The detection limit was 2.5 pg/mL.

### 4.6. Statistical Analysis

The normality was assessed using the Shapiro–Wilk test. The data from the healthy controls and patient groups were first analyzed by the Kruskall–Wallis test. The statistical comparisons of data between different pathologies and healthy controls were performed using the Mann–Whitney U test. Correlations were assessed using Spearman’s rank correlation coefficient. Differences were considered significant when *p* values were <0.05. All of the statistical analysis of data was carried out with the SPSS 15.0 software (Chicago, IL, USA).

## Figures and Tables

**Table 1:** Demographic and main clinical features of pAPS, SLE, and HC.

	HC	pAPS	SLE
Number of patients, *n*	21	37	11
Age (mean ± SD)	40.3 ± 11.8	36.7 ± 9.9	32.8 ± 13.1
Females, *n* (%)	15 (71.4)	30 (81.1)	11 (100)
Clinical manifestations associated with APS, *n* (%)	0	37 (100)	0
- Obstetrical events, *n* (%)	-	17 (45.9)	-
- Arterial thrombosis, *n* (%)	-	12 (32.4)	-
- Venous thrombosis, *n* (%)	-	8 (21.6)	-
Positive aPL Serology, *n* (%)	0	37 (100)	6 (54.5)
- Positivity for one Ab, *n* (%)	-	19 (51.3)	4 (36.3)
- Positivity for two Ab, *n* (%)	-	12 (32.4)	0 (0)
- Positivity for three Ab, *n* (%)	-	6 (16.2)	2 (18.2)
Serological profile			
- aCL, *n* (%)	-	29 (78.4)	5 (45.5)
- aβ2GPI, *n* (%)	-	17 (45.9)	2 (18.2)
- Lupus Anticoagulant, *n* (%)	-	15 (40.5)	3 (27.3)
Peripheral blood cells			
Lymphocytes (cells/mm^3^)	2211.00 (1838.0–2915.0)	1852.00 (1545.0–2590.5)	1721.00 (1082.0–2475.0)
CD19^+^ B cells (cells/mm^3^)	226.50 (181.75–341.25)	185.00 (140.50–296.00)	112.00 (87.00–325.00)
CD19^+ ^B cells (%)	11.50 (8.25–13.75)	10 (7.00–12.50)	10 (7.00–12.00)
Treatment			
- Antiplatelet, *n* (%)		23 (62.2)	4 (36.4)
- Anticoagulant, *n* (%)		16 (43.2)	1 (9.1)
- Corticosteroids, *n* (%)		4 (10.8)	3(27.3)
- Antimalarials, *n* (%)		5 (13.5)	9 (81.8)
- Immunosuppresors, *n* (%)		0 (0)	2 (18.2)

pAPS: primary antiphospholipid syndrome; aPL: antiphospholipid; SLE: systemic lupus erythematosus; SD: standard deviation; aCL: anticardiolipin; aB2GPI: anti beta2glycoprotein; Ab: antibody; HC: healthy control.

## Supplementary Materials

The following are available online at http://www.mdpi.com/1422-0067/19/2/589/s1.
