# Supplementary material for: Peripheral B-Cell Subset Distribution in Primary Antiphospholipid Syndrome
Source: Int J Mol Sci. 2018 Feb 16;19(2):589. doi: 10.3390/ijms19020589 (PMC5855811; doi:10.3390/ijms19020589)
Supplement: Supplementary file 1 [file ijms-19-00589-s001.pdf]

Table S1. B cells disturbances in APS according to previous literature.

Author	N of patients	Material and Methods	B cells	Results
Carbone (1)<br>2009	36 women with pAPS : 26 obstetric APS without thrombosis (A1) and 10 obstetric APS with thrombosis (A2)<br>36 women with spontaneousrecurrent abortion without aPL (B)<br>36 healthy parous women (C)<br>36 healthy non parous women (D)	- Three colour inmunophenotyping of peripheral blood lymphocytes (EDTA). Whole Blood Lysis technique.<br>- mAb: CD27 / IgD / CD19<br>- Acquisition: 5000 events in a lymphocyte gate. FACScan.	- Naïve<br>(CD19+ CD 27- IgD+)<br>- Memory B cells<br>(class switched CD 19+CD27+IgD- and non-class-switched CD 19+ CD27+IgD+)	- No differences in the number and frequency of CD19+ B cells among groups<br>A2: increased frequency and absolute numbers of naïve cells compared to A1 and controls.<br>A2: lower percentage of memory class-switched and non-class switched than A1 and controls.<br>Higher naïve/memory class-switched ratio in A2 compared with controls.
Dal Ben (2)<br>2013	20 pAPS and 20 HC<br>(85%females)	PBMCs resuspended in RPMI+FBS and treated with FC blocking. Immunophenotyping with CD3 and CD19.<br>- Acquisition 20000 cells. FACs Canto II. Software analysis: Flow Jo.	CD 3-CD19+ B cells	- Lower percentages of total lymphocytes in APS than controls<br>- Lower percentages of CD3-CD19+ B cells in APS than controls.
Dal Ben(3)<br>2014	25 APS related SLE and 25 HC<br>96 % females	No data	CD3- CD19+ B cells	- No differences of total lymphocytes<br>- Lower numbers of CD3-CD19+ B cells in APS related SLE than in HC
Simonin(4)<br>2017<br>.	11 pAPS VTE<br>(54.5 % females)<br>11 nonAPS VTE<br>49 controls (OA)<br>11 SLE/11 RA/11pSS	- Four colour inmunophenotyping of peripheral blood lymphocytes (EDTA). Whole Blood Lysis technique.<br>- mAb: CD5/ CD27 / IgD / CD19/CD 38/ CD24/<br>- Acquisition 175.000 cells Gating lymphocytes.<br><br>- Beckman Coulter flow cytometer. (Navios + Kaluza)	B1 cells (CD19+ CD 5+)<br>Transitional B cells (CD19+ CD24++ CD 38++)<br>Naïve B cells (CD19+ IgD+CD27-)<br>Unswitched memory B cells (CD 19+ IgD+ CD27+)<br>Switched memory B cells (CD19+ IgD- CD27+)<br>Double negative B cells (CD19+IgD-CD27-)	- No differences in the frequency of CD19+B cells in pAPS VTE compared to controls and SLE<br>- Increase in percentage of B1 cells in pAPS VTE compared to non-APS VTE but not with controls.<br>- Increase in number and frequency of transitional B cells in pAPS VTE compared to nonAPS VTE and controls.<br>- Increase number but not frequency of naïve B cells in pAPSVTE compared to nonAPS VTE. No differences compared to controls.

				- No differences in the frequency of unswitched compared to nonAPS VTE and controls<br>Decrease number and percentage of SM B cells in pAPS VTE compared to controls.<br>No differences in the frequency of CD19+B cells in pAPS VTE compared to controls and SLE<br>Increase in number and frequency of naïve B cells in pAPS VTE compared to SLE and RA.
Present study	37 pAPS (17 Obstetric pAPS, 20 Trombotic pAPS)<br>11 SLE<br>21 HC	- Six colours immunophenotyping of PBMCs<br>- mAb: CD19, CD5, CD10, IgD , CD27, CD24 and CD38, IgM.<br>Immunophenotyping500.000 cells/sample.<br>- Acquisition 50000 cells. FACs Canto II.<br>Software analysis: FACS Diva<br>Gating CD19+ cells<br>Absolute numbers and % of lymphocytes by Multitest TruCount method (BD)	Immature($CD19^{+}CD5^{+}CD10^{+}$ $CD27^{-}IgD^{++}$ $CD24^{hi}$ $CD38^{hi}$).<br>- Naïve ($CD19^{+}$ $CD5^{+/-}$ $CD10^{-}$ $CD27^{-}IgD^{+}$ $CD24^{int}$ $CD38^{low}/^{-}$).<br>- Non-switched memory ($CD19^{+}$ $CD27^{+}IgD^{+}$ $CD38^{-}$ IgM^{+}),<br>- Switched memory ($CD19^{+}$ $CD27^{+}IgD^{-}$ $CD38^{+}IgM^{+/-}$),<br>- Doubles negatives ($CD19^{+}$ $CD27^{-}IgD^{-}$ $CD24^{-}$ $CD38^{-/+}$ IgM^{-})<br>- Plasma cells ($CD19^{lo}$ $CD27^{hi}IgD^{-}$ $CD38^{hi}$ $CD138^{-/+}$).	- No differences in the number and frequency of CD19+ B cells among three groups.<br>- Lower percentage ofimmature and naïve B cells in pAPS than SLE, especially in obstetric pAPS .No differences between thrombotic pAPS and SLE or HC.<br>- Higher frequency of non-switched memory cells in obstetric and thrombotic pAPS than SLE.No differences between pAPS and HC.<br>- No differences in frequencies of switched memory and plasma cells among three groups.<br>- SLE increased frequency of naive and decreased frequency of non switched memory compared to HC<br>- Higher double negative memory B cells in SLE than HC but not in pAPS

pAPS: primary antiphospholipid syndrome, opAPS: obstetric primary antiphospholipid syndrome, SLE: systemic lupus erythematosus, HC: healthy controls, OA: ostheoarthritis, RA: rheumatoid arthritis, pSS: primary Sjogren syndrome, VTE: venous thromboembolism.

Reference List

(1) Carbone J, Gallego A, Lanio N, Navarro J, Orera M, Aguaron A et al. Quantitative abnormalities of peripheral blood distinct T, B, and natural killer cell subsets and clinical findings in obstetric antiphospholipid syndrome. J Rheumatol 2009; 36(6):1217-1225.

(2) Dal Ben ER, Do Prado CH, Baptista TS, Bauer ME, Staub HL. Decreased levels of circulating CD4+CD25+Foxp3+ regulatory T cells in patients with primary antiphospholipid syndrome. J Clin Immunol 2013; 33(4):876-879.

(3) Dal Ben ER, Do Prado CH, Baptista TS, Bauer ME, Staub HL. Patients with systemic lupus erythematosus and secondary antiphospholipid syndrome have decreased numbers of circulating CD4(+)CD25(+)Foxp3(+) Treg and CD3(-)CD19(+) B cells. Rev Bras Reumatol 2014; 54(3):241-246.

(4) Simonin L, Pasquier E, Leroyer C, Cornec D, Lemerle J, Bendaoud B et al. Lymphocyte Disturbances in Primary Antiphospholipid Syndrome and Application to Venous Thromboembolism Follow-Up. Clin Rev Allergy Immunol 2016; 3878: 1-14.

Table S2. Imunophenotyopic characterization of B cell subsets.

**Immatures**	$CD19^{+}$ $CD5^{+}$ $CD10^{+}$ $CD27^{-}$ IgD^{++} $CD24^{hi}$ $CD38^{hi}$
**Naïve**	$CD19^{+}$ $CD5^{+/-}$ $CD10^{-}$ $CD27^{-}$ IgD^{+} $CD24^{int}$ $CD38^{-}$
**Non switched memory**	$CD19^{+}$ $CD27^{+}$ IgD^{+} $CD38^{-}$ IgM^{+}
**Switched memory**	$CD19^{+}$ $CD27^{+}$ IgD^{-} $CD38^{-/+}$ $IgM^{+/-}$
**Doubles negatives**	$CD19^{+}$ $CD27^{-}$ IgD^{-} $CD24^{-}$ $CD38^{-/+}$ IgM^{-}
**Plasma cells**	$CD19^{lo}$ $CD27^{hi}$ IgD^{-} $CD38^{hi}$ $CD138^{-/+}$
